# An Exponential-Cum-Sine-Type Hybrid Imputation Technique for Missing Data

**DOI:** 10.1155/2021/4845569

**Published:** 2021-12-03

**Authors:** D. Bhattacharyya, G. N. Singh, Taghreed M. Jawa, Neveen Sayed-Ahmed, Awadhesh K. Pandey

**Affiliations:** ^1^Department of Mathematics & Computing, Indian Institute of Technology (ISM), Dhanbad-826 004, Jharkhand, India; ^2^Department of Mathematics and Statistics, College of Science, Taif University, P.O. Box 11099, Taif 21944, Saudi Arabia; ^3^Department of Mathematics, School of Physical Sciences, DIT University, Dehradun, Uttarakhand 248009, India

## Abstract

In this study, a new exponential-cum-sine-type hybrid imputation technique has been proposed to handle missing data when conducting surveys. The properties of the corresponding point estimator for population mean have been examined in terms of bias and mean square errors. An extensive simulation study using data generated from normal, Poisson, and Gamma distributions has been conducted to evaluate how the proposed estimator performs in comparison to several contemporary estimators. The results have been summarized, and discussion regarding real-life applications of the estimator follows.

## 1. Introduction

The impracticality of measuring the entire population for any realistic project due to budgetary, time, or other constraints makes sampling indispensible for any field of study [1–12]. The widespread applications of acceptance sampling in various industries for manufacturing and other processes have been noted for a considerable period of time. Sampling can also be applied to obtain vital information on the chief characteristics of items ranging from electrical appliances to machine parts such as screws and bolts, automobiles, and computer parts such as chip. In addition, many environmental problems involve physical, geographical, economical, and other characteristics which need to be estimated prior to data analysis, model formulation, and predictions. Studies related to the amount of rainfall received annually in a flood-prone area, the quality of drinking water near an industrial zone, the soil quality of an agricultural land, etc. are some instances where estimation of mean, median, variance, and other statistics is essential. Such information can be collected via sample surveys [4, 6, 7, 9, 13].

Missing data is a universal occurrence in sample surveys, leading to a decline in data quality and complications in making inferences. It is pivotal for survey statisticians to factor in the stochastic nature of incomplete data. This brings forth the question of what assumptions have to be made or which techniques have to be employed to handle the problem of ignorability of completeness mechanism. The mechanisms of missing data have been studied in detail in [9, 13], among others. Three missing data mechanisms are mostly of interest in the survey literature, namely, missing completely at random (MCAR), missing at random (MAR), and missing not at random (MNAR). MCAR is said to occur when data is missing randomly or by chance, MAR occurs when the missingness does not depend on the variable under study (which may be unobserved), but on some other variables (which is fully observed), and MNAR occurs when missingness depends on the variable under study.

Numerous statistical methods have been devised over the years to overcome the problem of missing data. Subsampling of nonrespondents in surveys via mail questionnaire was pioneered in [8]. Another commonly used method is imputation, in which the missing values are filled in by a suitable function of the available values, to ensure the structural completeness of the sample before analysis begins. Popular imputation techniques include mean imputation, regression imputation, hot deck imputation, cold deck imputation, and nearest neighbor method. Imputation techniques in the survey literature are from [3, 5, 14–21], among others. Some recent works in the area of imputation and estimation of population mean have been done in [22–29] and others.

Information from an auxiliary variable can be utilized to provide an improved estimate for population characteristics. Such information may be readily available as secondary data from previous surveys or census or may be collected during the survey procedure at little to no additional cost. Some examples of such auxiliary information include the lifetime of a previous batch of bulbs when studying the life of a current lot of bulbs, the speed of cars when studying the mileage of cars, etc.

In this manuscript, a new exponential-cum-sine-type hybrid imputation technique and corresponding point estimator have been proposed for estimation of population mean. Motivation for this estimator, its properties, and its uses have been discussed in the subsequent sections. The manuscript is henceforth divided into the following sections: Section 2 introduces the sample structure and notations used in the manuscript.  Section 3 discusses some conventional estimators of population mean.  Section 4 discusses the proposed estimator, including its existence, consistency, properties, and implementation in R. The simulation study has been presented in Section 5, the results and discussion in Section 6, and the conclusions in Section 7.

## 2. Sample Structure and Notations Used

Let the character of interest be denoted by *Y*. We consider the scenario in which complete information on a correlated auxiliary variable *X* is available to the survey statisticians and its population mean is known.

The sample structure and the notations used henceforth have been introduced in Table 1.

## 3. Some Conventional Estimators

Before the proposed estimator is introduced, it is important to examine some existing estimators for population mean and study their strengths and limitations. A few such estimators have been discussed in this section.

The mean estimator is a simple and traditional estimator, which makes use of the average of the responses to provide an estimate of the population mean. The ratio estimator tries to make an improvement over the mean estimator by incorporating auxiliary information into a correlated variable. Various other estimators that make innovative use of auxiliary information have been proposed, for instance, the estimator proposed in [30], regression-type estimators proposed in [10], and exponential type estimators in [31], among others.

The structures of some of these estimators have been given in Table 2, while the expressions for their respective variances (V) or mean square errors (MSEs) have been given in Table 3.

It is to be noted that most conventional estimators make use of simple functional forms, such as linear combinations, exponential functions, and chains. Combination of multiple mathematical functions is rarely seen. This can be attributed to computational limitations associated with such functions. However, with the advent of supercomputers and improvement in computational powers, such obstructions have been eliminated. It is worth exploring whether combinations of mathematical functions produce better estimates than traditional estimators. This has been the motivation behind the construction of the proposed estimator.

Two such functions have been used, namely, the exponential and sine functions. Such particular functions were selected based on their use in real-life situations. The exponential function is usually used to model growth and decay observed in nature, such as growth and decay of microorganisms like bacteria, human population, spread of pandemics, and compound interests. Sine function is commonly utilized for the purpose of modeling natural phenomena which are periodic in nature, such as sound waves, light waves, tides, sunlight intensity, and average temperature variations through the year, as well as ballistic trajectories, electrical currents, and GPS locations.

## 4. Formulation of the Proposed Estimator

Let *y*_*i*_ and *x*_*i*_ be the values of *Y* and *X*, respectively, for the *i*^th^ unit in the population. The following imputation method may be suggested to deal with the problem of missing data:

(1) $$\begin{array}{c} y_{\cdot i}=\left\{\begin{array}{cc} y_{i}, & \mathrm{if}\;i\in R,\\ \frac{n}{n-r}x_{i}\exp\left[\frac{\sin\left({\bar{x}}_{n}\right)-\sin\left({\bar{x}}_{r}\right)}{1+\sin\left({\bar{x}}_{n}\right)+\sin\left({\bar{x}}_{r}\right)}\right]-\frac{r}{n-r}{\bar{y}}_{r}, & \mathrm{if}\;i\in R^{c}. \end{array}\right. \end{array}$$

The point estimator under an imputation method is given in

(2) $$\begin{array}{c} T=\frac{1}{n}\sum_{i\in S}y_{\cdot i}=\frac{1}{n}\left[\sum_{i\in R}y_{\cdot i}+\sum_{i\in R^{c}}y_{\cdot i}\right]. \end{array}$$

Using equation (2), under the imputation outlined in equation (1), the expression for the point estimator of $\bar{Y}$ is obtained as

(3) $$\begin{array}{c} T={\bar{y}}_{r}\exp\left[\frac{\sin\left({\bar{x}}_{n}\right)-\sin\left({\bar{x}}_{r}\right)}{1+\sin\left({\bar{x}}_{n}\right)+\sin\left({\bar{x}}_{r}\right)}\right]. \end{array}$$

### 4.1. Existence and Consistency of the Estimator

It is important to specify the domain of values for which an estimator exists, so that survey statisticians or those working in the field can determine whether an estimator can be reasonably used in a practical scenario.

The given estimator consists of two major functions: the trigonometrical function sin() and the exponential function exp(). Both sin(*x*) and exp(*x*) exist in ∀*x* ∈ *ℝ*, so *y*_·*i*_ and *T* exist in ∀*x* ∈ *ℝ*.

Hence, the proposed estimator can be used for all real values of the characters under study. For real-world scenarios, most, if not all, characters of interest take only real values. For example, measurements such as length, breadth, height, weight, diameter, currencies, and number of an item do not take nonreal values. Hence, the proposed estimator can be used in all practical scenarios.

It is to be noted that the structure of the estimator is consistent for large sample approximations. As *n*⟶*∞*, ${\bar{y}}_{r}⟶\bar{Y}$, ${\bar{x}}_{r}⟶\bar{X}$, ${\bar{x}}_{n}⟶\bar{X}$, and exp(0)=1. Hence, $T⟶\bar{Y}$.

### 4.2. Properties of the Proposed Estimator

The “goodness” of an estimator can be measured in terms of various properties. Two such properties, namely, bias and mean squared error (MSE), have been explored here. The bias gives an idea about the expected deviation from the true value of a parameter, while MSE deals with the degree of spread. The expressions for the same have been derived under large sample assumptions up to the first order of approximations. Some transformations involving error terms have been used for the purpose, indicated as follows:

(4) $$\begin{array}{c} {\bar{y}}_{r}=\bar{Y}\left(1+\eta_{0}\right),\\ {\bar{x}}_{r}=\bar{X}\left(1+\eta_{1}\right),\\ {\bar{x}}_{n}=\bar{X}\left(1+\eta_{2}\right),\\ \theta_{1}=\left(\frac{1}{r}-\frac{1}{N}\right),\\ \theta_{2}=\left(\frac{1}{n}-\frac{1}{N}\right),\\ \theta_{3}=\left(\frac{1}{r}-\frac{1}{n}\right). \end{array}$$

The error terms have the following expectations:

(5) $$\begin{array}{c} E\left(\eta_{0}\right)=E\left(\eta_{1}\right)=E\left(\eta_{2}\right)=0,\\ E\left(\eta_{0}^{2}\right)=\theta_{1}C_{Y}^{2},\\ E\left(\eta_{1}^{2}\right)=\theta_{1}C_{X}^{2},\\ E\left(\eta_{2}^{2}\right)=\theta_{2}C_{X}^{2},\\ E\left(\eta_{0}\eta_{1}\right)=\theta_{1}\rho C_{Y}C_{X},\\ E\left(\eta_{1}\eta_{2}\right)=\theta_{2}C_{X}^{2},\\ E\left(\eta_{0}\eta_{2}\right)=\theta_{2}\rho C_{Y}C_{X}. \end{array}$$

To obtain the expressions for bias and MSE, in the first step, algebraic expansion of the expression of the estimator given in equation (3) is done, using the following Taylor's series:

1. sin(*x*)=*x* − (*x*^3^/3!)+(*x*^5^/5!) − (*x*^7^/7!) − ⋯
2. exp(*x*)=1+*x*+(*x*^2^/2!)+(*x*^3^/3!)+(*x*^4^/4!)+⋯
3. (1+*x*)^−1^=1 − *x*+*x*^2^ − *x*^3^+⋯

The estimator takes the following form:

(6) $$\begin{array}{c} T={\bar{y}}_{r}\left[1+{\bar{x}}_{n}-{\bar{x}}_{r}+2{\bar{x}}_{r}^{2}-2{\bar{x}}_{n}{\bar{x}}_{r}\right]. \end{array}$$

In the second step, the transformations in equation (4) are applied to equation (6) to obtain the following form of the estimator:

(7) $$\begin{array}{c} T=\bar{Y}\left[1+\bar{X}\left(\eta_{2}-\eta_{1}\right)+\eta_{0}\eta_{2}{\bar{X}}^{2}\left(\eta_{1}-\eta_{2}+\eta_{1}^{2}-\eta_{1}\eta_{2}+\eta_{0}\eta_{1}-\eta_{0}\eta_{2}\right)+\bar{X}\left(\eta_{0}\eta_{2}-\eta_{0}\eta_{1}\right)\right]. \end{array}$$

Hence, $T-\bar{Y}=\bar{Y}\left[\bar{X}\left(\eta_{2}-\eta_{1}\right)+\eta_{0}\eta_{2}{\bar{X}}^{2}\left(\eta_{1}-\eta_{2}+\eta_{1}^{2}-\eta_{1}\eta_{2}+\eta_{0}\eta_{1}-\eta_{0}\eta_{2}\right)+\bar{X}\left(\eta_{0}\eta_{2}-\eta_{0}\eta_{1}\right)\right]$.

Expectations taken on both sides and use of the expected values of *η*_*i*_, *i*=0,1,2, yield the expectations for bias *B*(.) and MSE (*M*(.)), obtained up to the first order of approximations of the estimators *T*_*i*_, *i*=1,2,…, 6, as follows:

(8) $$\begin{array}{c} B\left(T\right)=E\left(T-\bar{Y}\right)=\bar{Y}\left[\left(2{\bar{X}}^{2}-\bar{X}\right)\theta_{3}\rho C_{Y}C_{X}-2{\bar{X}}^{2}\theta_{2}C_{X}^{2}\right], \end{array}$$

(9) $$\begin{array}{c} M\left(T\right)=E{\left(T-\bar{Y}\right)}^{2}=\theta_{1}S_{Y}^{2}+{\bar{Y}}^{2}\theta_{3}\left[C^{2}C_{X}^{2}+2\;C\rho C_{Y}C_{X}\right], \end{array}$$

where $C=2{\bar{X}}^{2}-\bar{X}$.

### 4.3. Implementation in R

In the current day and age, most computations are carried out using a suitable software environment. The following R [32] code snippet has been developed to carry out the proposed imputation on a data set of interest and calculate the value of the corresponding point estimator:

  #Import data of respondents from file
 
*df*resp < −read.table (file.choose())
  #Import data of nonrespondents from file
 
*df*nonresp < −read.table (file.choose())
 
*xr*bar = mean (*df*resp[, 1])
 
*yr*bar < −mean (*df*resp[, 2])
 
*x*barnonresp = mean (*df*nonresp[, 1])
 
*r* = *n*row (*df*resp) #no. of respondents
  nonresp = *n*row (*df*nonresp) #no. of nonrespondents
 
*n* = *r* + nonresp #sample size
 
*xn*bar=(*r∗xr*bar + nonresp*∗x*barnonresp)/*n*
  num = sin(*xn*bar) − sin(*xr*bar)
  den = 1 + sin(*xn*bar)+sin(xrbar)
  #imputation
 
*t* < −*c*()
  for (*i* in 1 : (*n* − *r*))
  {
 
*t*[*i*] = *n*/(*n* − *r*)*∗x*[*i*]*∗*exp(num/den) − *r*/(*n* − *r*)*∗yr*bar
  }
  #point estimation
  est = *yr*bar*∗*exp(num/den)

## 5. Simulation Study

Before an estimator can be used in practical scenarios, its performance must be examined, in terms of its properties. To this end, the bias of the estimator is calculated and the MSE is compared with that of the contemporary estimators given in Table 2 in terms of percentage relative efficiencies (PREs).

The PREs of the estimator with respect to the contemporary estimators are defined as follows:

(10) $$\begin{array}{c} {\text{PRE}}_{1}=\frac{V\left({\bar{y}}_{m}\right)}{M\left(T\right)}\times 100,\\ {\text{PRE}}_{2}=\frac{M\left({\bar{y}}_{\text{RAT}}\right)}{M\left(T\right)}\times 100,\\ {\text{PRE}}_{3}=\frac{M\left(T_{\text{TSS}}\right)}{M\left(T\right)}\times 100,\\ {\text{PRE}}_{4}=\frac{M\left(T_{\text{SMKK}}\right)}{M\left(T\right)}\times 100,\\ {\text{PRE}}_{5}=\frac{M\left(T_{{\text{KC}}_{1}}\right)}{M\left(T\right)}\times 100,\\ {\text{PRE}}_{6}=\frac{M\left(T_{{\text{KC}}_{2}}\right)}{M\left(T\right)}\times 100,\\ {\text{PRE}}_{7}=\frac{M\left(T_{{\text{KC}}_{3}}\right)}{M\left(T\right)}\times 100, \end{array}$$

where the expression for the MSE of the proposed estimator *T* is given in equation (9), while that of the contemporary estimators is given in Table 3.

Using R [32], an extensive simulation study has been carried out on sufficiently large fictitious populations to compute the bias and the PREs defined above. Data is generated from three different probability distributions, namely, normal and Gamma distributions (continuous distributions) and Poisson distribution (discrete distribution). Some important properties of the distributions have been summarized in Table 4. Such distributions are chosen based on their occurrence in real-life situations.

Data from normal distribution is rampant in nature. It can be used to model heights of individuals, test scores of students, blood pressure, daily returns of any particular stock, weights of items produced by a manufacturing process, etc. Poisson distribution can be used to model the probability that a given number of events occur in a specific time interval, for example, the number of insurance claims filed per month, the number of network failures occurring per week, and the number of bulbs manufactured per minute. It also finds use by medical statisticians, such as for estimating the number of births that may be expected on a particular night, the number of patients with an infectious disease arriving at a clinic within a given hour, the number of mutations on a given strand of DNA per time unit, etc. Gamma distribution can be used for modeling wait time, reliability, service time in queuing theory, etc. For example, it can be used to model the amount of rainfall that accumulates in a given reservoir, the flow of items through manufacturing as well as distribution processes, the size of loan defaults, etc. Thus, these three distributions are chosen based on their importance in practical scenarios.

It is seen through trial and error that the estimator performs well when *X* and *Y* take small values and the variation in *X* is greater than that in *Y*.

The steps of the simulation are as follows:

1. The sizes of the population, the sample, and the responding part of the sample are defined. For the purpose of the study, sufficiently large values of *N*=100000, *n*=40000, and *r*=35000 have been chosen.
2. The parameters of the population are defined.
3. Simulation is conducted for various values of *ρ*. For the purpose of the study, *ρ* in the range (0.1, 0.9); i.e., positively correlated variable *X* is considered.

The results of the simulation study related to the PREs have been presented in Tables 5–11, while the biases have been presented in Table 12.

## 6. Results and Discussion

The simulation study enables us to study the behavior of the proposed estimator under various scenarios involving various values of parameters. The chief conclusions are as follows:

1. From the values of PRE_1_ in Table 5, it is seen that the proposed estimator is more efficient than ${\bar{y}}_{m}$ for all values of *ρ* for normal data and for *ρ* ∈ (0.2, 0.9) for Gamma and Poisson data for the various values of response rates.
2. It is seen that the proposed estimator performs better than ${\bar{y}}_{\text{RAT}}$ for all values of *ρ* for normal and Gamma data and for *ρ* ∈ (0.1, 0.8) for Poisson data for the various values of response rates from the values of PRE_2_ in Table 6.
3. From the values of PRE_3_ in Table 7, it is seen that the proposed estimator dominates *T*_TSS_ for all values of *ρ* for normal data and for *ρ* ∈ (0.1, 0.7) for Gamma and Poisson data for the various values of response rates.
4. The values of PRE_4_ in Table 8 show that the proposed estimator is more efficient than *T*_SMKK_ for all values of *ρ* for normal data and for *ρ* ∈ (0.1, 0.7) for Gamma and Poisson data for the various values of response rates.
5. In Table 9, the values of PRE_5_ show that the proposed estimator performs better than *T*_KC_*A*__ for all values of *ρ* and for the various values of response rates for normal, Gamma, and Poisson data.
6. From the values of PRE_6_ in Table 10, it is seen that the proposed estimator dominates *T*_KC_*B*__ for all values of *ρ* and for the various values of response rates for normal, Gamma, and Poisson data.
7. It is seen that the proposed estimator is more efficient than *T*_KC_*C*__ for all values of *ρ* and for the various values of response rates for normal, Gamma, and Poisson data from the values of PRE_7_ in Table 11.
8. From Table 12, it is seen that the estimator is negatively biased. The bias is negligible, being of the order 10^−5^ and 10^−7^ for various values of the parameter *ρ* and for various response rates, and hence, bias correction is not needed.

## 7. Conclusion

The following trend in the PREs is noticed from the tables: PRE_1_ increases with the increase in value of *ρ*, while PRE_2_, PRE_3_, PRE_4_, PRE_5_, PRE_6_,  and PRE_7_ decrease with the increase in value of *ρ*.

The proposed estimator is seen to be consistent, exists for all real values of parameters, has negligible bias, and is more efficient than 7 other contemporary estimators. Hence, the proposed estimator may be recommended for use in field work.

## Figures and Tables

**Table 1:** Sample structure and notations.

Structure	Size
Population	*N*
Sample	*N*
Respondents	*R*
Nonrespondents	*N* − *r*

Characteristic	Notation
The population mean of *Y*	$\bar{Y}$
The population mean of *X*	$\bar{X}$
The sample mean of *Y* based on the responding part of the sample	${\bar{y}}_{r}$
The sample mean of *X* based on the responding part of the sample	${\bar{x}}_{r}$
The sample means of *X*, respectively, based on the entire sample	${\bar{x}}_{n}$
The correlation coefficient between *X* and *Y*	*ρ*
The population mean square of *X*	*S*_*X*_^2^
The population mean square of *Y*	*S*_*Y*_^2^
The coefficient of variation of *X*	*C*_*X*_
The coefficient of variation *Y*	*C*_*Y*_

**Table 2:** Structures of some well-known estimators.

Estimator	Notation used	Structure
Mean estimator	${\bar{y}}_{m}$	${\bar{y}}_{r}$
Ratio estimator	${\bar{y}}_{\text{RAT}}$	${\bar{y}}_{r}\left({\bar{x}}_{n}/{\bar{x}}_{r}\right)$
Kadilar and Cingi [10] estimator A	*T*_KC_*A*__	$\left({\bar{y}}_{r}+b\left(\bar{X}-{\bar{x}}_{n}\right)/{\bar{x}}_{n}\right)\bar{X}$
Kadilar and Cingi [10] estimator B	*T*_KC_*B*__	$\left({\bar{y}}_{r}+b\left(\bar{X}-{\bar{x}}_{r}\right)/{\bar{x}}_{r}\right)\bar{X}$
Kadilar and Cingi [10] estimator C	*T*_KC_*C*__	$\left({\bar{y}}_{r}+b\left({\bar{x}}_{n}-{\bar{x}}_{n}\right)/{\bar{x}}_{r}\right)\bar{X}$
Toutenberg and Srivastava [30] estimator	*T*_TSS_	${\bar{y}}_{r}+\left(r/n\right)\left({\bar{y}}_{r}/{\bar{x}}_{n}\right)\left({\bar{x}}_{n}-{\bar{x}}_{r}\right)$
Singh et al. [31]	*T*_SMKK_	${\bar{y}}_{r}\left({\bar{x}}_{n}/{\bar{x}}_{r}\right)\exp\left[\bar{X}-{\bar{x}}_{r}/\bar{X}+{\bar{x}}_{r}\right]$

**Table 3:** MSEs of some well-known estimators.

Estimator	Variance (V) or mean square error (MSE)
${\bar{y}}_{m}$	$V\left({\bar{y}}_{m}\right)=\theta_{1}S_{Y}^{2}$
${\bar{y}}_{\text{RAT}}$	$\text{MSE}\left({\bar{y}}_{\text{RAT}}\right)=\theta_{2}S_{Y}^{2}+\theta_{3}\left(S_{Y}^{2}+R_{1}^{2}S_{X}^{2}-2R_{1}\rho S_{Y}S_{X}\right)$
*T*_KC_*A*__	MSE(*T*_KC_*A*__)=((1/*r*) − (1/*N*))*S*_*Y*_^2^+((1/*n*) − (1/*N*))*S*_*X*_^2^(*R*_1_^2^ − *B*^2^)
*T*_KC_*B*__	MSE(*T*_KC_*B*__)=((1/*r*) − (1/*N*))(*S*_*Y*_^2^ − *BS*_*YX*_+*R*^2^*S*_*X*_^2^)
*T*_KC_*C*__	MSE(*T*_KC_*C*__)=((1/*r*) − (1/*N*))*S*_*Y*_^2^+((1/*r*) − (1/*N*))((*R*+*B*)^2^*S*_*X*_^2^ − 2(*R*+*B*)*S*_*XY*_)
*T*_TSS_	$\text{MSE}\left(T_{\text{TSS}}\right)=\left(\left(1/r\right)-\left(1/N\right)\right)S_{Y}^{2}+{\bar{Y}}^{2}\left(\left(1/r\right)-\left(1/n\right)\right)\left(r/n\right)\left(\left(r/n\right)C_{X}^{2}-2\rho C_{Y}C_{X}\right)$
*T*_SMKK_	$M\left(T_{\text{SMKK}}\right)={\bar{Y}}^{2}\left[\left(\left(1/r\right)-\left(1/N\right)\right)\left(C_{Y}^{2}+\left(9/4\right)C_{X}^{2}-3\rho C_{Y}C_{X}\right)+2\left(\left(1/n\right)-\left(1/N\right)\right)\left(\rho C_{Y}C_{X}-C_{X}^{2}\right)\right]$
	Where $R_{1}=R=\left(\bar{Y}/\bar{X}\right)$, *B*=*S*_*XY*_/*S*_*X*_^2^

**Table 4:** Some properties of normal, Poisson, and Gamma distributions.

Distribution	Normal
Parameters	*μ*, *σ*^2^
Pdf	$f\left(x\right)=\left(1/\sigma \sqrt{2\pi }\right)\exp\left[-\left({\left(x-\mu \right)}^{2}/2\sigma^{2}\right)\right],\;-\infty <x<\infty$
Mean *E*(*X*)	*μ*
Variance *V*(*X*)	*σ* ^2^

Distribution	Poisson
Parameter	*λ* > 0
Pmf	*f*(*x*)=*λ*^*x*^*e*^−*λ*^/*x*!
Mean *E*(*X*)	*λ*
Variance *V*(*X*)	*λ*

Distribution	Gamma
Parameters	*α*, *λ*
Pdf	$f\left(x\right)=\left\{\begin{array}{cc} \lambda^{\alpha }x^{\alpha -1}e^{-\lambda x}/\Gamma \left(x\right), & \mathrm{if}\;x>0,\\ 0, & \text{otherwise} \end{array}\right.$
Mean *E*(*X*)	*α*/*λ*
Variance *V*(*X*)	*α*/*λ*^2^

**Table 5:** Values of PRE_1_ when *ρ* ∈ (0.1, 0.2, 0.3, 0.4, 0.5, 0.6, 0.7, 0.8, 0.9) and response rates are 75%, 80%, 85%, 90%,  and 95% for data generated from normal, Gamma, and Poisson distributions.

*ρ*	PRE_1_ when response rate is				
	75%	80%	85%	90%	95%
	When data is generated from normal distribution				
0.1	100.134 4	100.110 6	100.085 5	100.058 7	100.030 3
0.2	101.457 1	101.196 9	100.922 4	100.632 3	100.325 4
0.3	102.640 5	102.164 5	101.664 4	101.138 3	100.584 3
0.4	103.563 8	102.916 5	102.238 9	101.528 5	100.783 1
0.5	104.411 4	103.604 8	102.762 9	101.883 2	100.963 2
0.6	106.314 4	105.142 8	103.928 1	102.667 8	101.359 4
0.7	108.313 4	106.747 4	105.135 1	103.474 6	101.763 7
0.8	107.626 8	106.197 5	104.722 5	103.199 5	101.626 2
0.9	109.266 4	107.508 4	105.704 6	103.853 2	101.952 4

	When data is generated from Gamma distribution				
0.1	99.298 0	99.421 1	99.552 1	99.691 6	99.840 6
0.2	99.950 0	99.958 9	99.968 2	99.978 1	99.988 7
0.3	100.587 7	100.483 5	100.373 2	100.256 3	100.132 1
0.4	101.385 6	101.138 3	100.877 3	100.601 5	100.309 5
0.5	102.425 1	101.988 6	101.529 7	101.046 7	100.537 5
0.6	103.616 2	102.959 2	102.271 4	101.550 6	100.794 3
0.7	105.340 6	104.357 1	103.333 8	102.268 4	101.158 1
0.8	106.962 2	105.664 0	104.321 1	102.931 2	101.491 7
0.9	109.279 9	107.519 1	105.712 6	103.858 5	101.955 0

	When data is generated from Poisson distribution				
0.1	99.970 1	99.975 3	99.980 9	99.986 9	99.993 2
0.2	100.248 2	100.204 3	100.157 8	100.108 4	100.055 9
0.3	100.602 0	100.495 2	100.382 2	100.262 5	100.135 3
0.4	100.937 7	100.770 9	100.594 7	100.408 1	100.210 2
0.5	101.536 9	101.262 3	100.972 6	100.666 6	100.343 0
0.6	101.946 9	101.597 9	101.230 2	100.842 6	100.433 1
0.7	102.653 3	102.174 9	101.672 3	101.143 7	100.587 1
0.8	103.225 6	102.641 4	102.028 9	101.386 1	100.710 6
0.9	104.123 4	103.371 2	102.585 2	101.763 1	100.902 3

**Table 6:** Values of PRE_2_ when *ρ* ∈ (0.1, 0.2, 0.3, 0.4, 0.5, 0.6, 0.7, 0.8, 0.9) and response rates are 75%, 80%, 85%, 90%,  and 95% for data generated from normal, Gamma, and Poisson distributions.

*ρ*	PRE_2_ when response rate is				
	75%	80%	85%	90%	95%
	When data is generated from normal distribution				
0.1	769.433 6	651.167 6	525.795 1	392.656 0	251.005 3
0.2	299.679 7	264.020 4	226.399 2	186.649 7	144.586 0
0.3	274.230 3	242.818 2	209.819 3	175.110 1	138.554 2
0.4	496.315 6	424.337 8	348.974 3	269.980 6	187.087 8
0.5	365.407 7	316.882 7	266.229 3	213.304 3	157.951 6
0.6	161.467 9	150.062 8	138.238 0	125.969 8	113.233 0
0.7	194.934 1	177.050 6	158.639 9	139.678 2	120.140 5
0.8	142.841 8	134.812 9	126.527 2	117.972 2	109.134 6
0.9	157.471 7	146.568 1	135.380 7	123.898 2	112.108 8

	When data is generated from Gamma distribution				
0.1	167.446 9	155.613 4	143.030 6	129.625 0	115.313 2
0.2	163.157 7	152.016 8	140.198 6	127.639 3	114.266 9
0.3	159.970 7	149.336 5	138.081 8	126.150 7	113.480 4
0.4	151.366 5	142.198 7	132.523 9	122.299 1	111.475 8
0.5	145.333 2	137.174 2	128.596 2	119.566 4	110.047 9
0.6	137.744 9	130.887 0	123.707 7	116.184 2	108.290 9
0.7	129.067 1	123.714 1	118.144 9	112.346 0	106.302 9
0.8	113.216 1	110.751 8	108.202 6	105.564 2	102.831 7
0.9	100.842 1	100.682 3	100.518 4	100.350 1	100.177 4

	When data is generated from Poisson distribution				
0.1	139.707 9	132.702 4	125.271 4	117.375 1	108.968 4
0.2	136.595 4	130.124 2	123.267 0	115.988 2	108.247 6
0.3	132.307 4	126.577 8	120.514 3	114.086 8	107.261 4
0.4	129.075 8	123.905 2	118.439 9	112.653 9	106.518 2
0.5	122.248 5	118.272 8	114.079 5	109.650 3	104.964 8
0.6	117.655 7	114.490 2	111.156 5	107.640 7	103.927 6
0.7	109.955 8	108.160 7	106.274 9	104.291 6	102.202 8
0.8	104.363 1	103.572 8	102.744 4	101.874 9	100.961 2
0.9	93.266 0	94.494 4	95.778 0	97.120 6	98.526 5

**Table 7:** Values of PRE_3_ when *ρ* ∈ (0.1, 0.2, 0.3, 0.4, 0.5, 0.6, 0.7, 0.8, 0.9) and response rates are 75%, 80%, 85%, 90%,  and 95% for data generated from normal, Gamma, and Poisson distributions.

*ρ*	PRE_3_ when response rate is				
	75%	80%	85%	90%	95%
	When data is generated from normal distribution				
0.1	470.654 1	448.598 9	405.082 4	335.811 6	235.944 6
0.2	205.929 6	200.477 9	188.554 3	168.842 5	139.873 1
0.3	188.801 9	184.937 7	175.366 8	158.912 2	134.271 4
0.4	303.819 3	294.193 0	271.676 3	233.719 8	177.522 6
0.5	229.191 9	224.740 8	211.484 9	187.620 1	151.176 6
0.6	120.640 2	122.286 4	121.653 5	118.156 1	111.164 6
0.7	133.264 1	135.276 3	133.806 8	128.030 1	117.071 0
0.8	103.726 6	108.071 5	110.493 9	110.391 4	107.121 9
0.9	105.568 7	111.201 2	114.246 5	113.939 4	109.473 9

	When data is generated from Gamma distribution				
0.1	137.107 3	135.015 2	130.737 8	123.827 1	113.774 4
0.2	134.024 1	132.235 6	128.394 0	122.072 7	112.790 1
0.3	131.435 3	129.955 5	126.514 3	120.695 9	112.033 5
0.4	125.754 8	124.791 5	122.129 5	117.396 2	110.175 1
0.5	121.186 1	120.746 3	118.779 8	114.934 1	108.818 8
0.6	115.379 1	115.641 5	114.583 8	111.873 7	107.146 3
0.7	108.611 5	109.731 4	109.758 0	108.377 1	105.247 9
0.8	97.570 0	99.957 8	101.676 9	102.455 0	102.000 3
0.9	88.167 8	91.827 8	95.109 0	97.750 4	99.477 3

	When data is generated from Poisson distribution				
0.1	121.737 3	120.509 3	118.000 0	113.948 4	108.059 8
0.2	119.469 0	118.492 9	116.324 8	112.714 2	107.379 0
0.3	116.387 1	115.749 6	114.042 9	111.031 3	106.449 9
0.4	113.897 9	113.565 1	112.251 2	109.728 2	105.740 3
0.5	108.956 1	109.183 7	108.621 6	107.062 5	104.275 0
0.6	105.457 8	106.119 4	106.113 7	105.242 8	103.286 8
0.7	99.941 6	101.235 9	102.075 0	102.282 5	101.663 1
0.8	95.626 7	97.480 5	99.022 0	100.082 7	100.477 0
0.9	87.555 7	90.392 2	93.207 4	95.855 8	98.178 2

**Table 8:** Values of PRE_4_ when *ρ* ∈ (0.1, 0.2, 0.3, 0.4, 0.5, 0.6, 0.7, 0.8, 0.9) and response rates are 75%, 80%, 85%, 90%,  and 95% for data generated from normal, Gamma, and Poisson distributions.

*ρ*	PRE_4_ when response rate is				
	75%	80%	85%	90%	95%
	When data is generated from normal distribution				
0.1	1916.780 1	1674.549 3	1417.763 3	1145.069 6	854.942 7
0.2	647.902 5	569.861 9	487.527 8	400.535 9	308.479 5
0.3	582.505 4	509.820 1	433.462 7	353.148 0	268.559 9
0.4	1197.061 4	1032.498 1	860.194 1	679.590 3	490.072 0
0.5	844.349 3	725.848 4	602.149 8	472.904 1	337.729 5
0.6	281.945 8	244.044 6	204.748 7	163.979 6	121.653 0
0.7	380.226 8	322.547 7	263.168 1	202.011 5	138.996 9
0.8	240.640 9	203.163 7	164.487 8	124.554 8	83.302 3
0.9	288.721 7	238.802 4	187.583 5	135.013 8	81.038 9

	When data is generated from Gamma distribution				
0.1	284.139 1	259.915 0	234.157 0	206.714 6	177.417 1
0.2	272.979 2	249.299 4	224.179 9	197.485 3	169.062 6
0.3	265.010 4	241.396 3	216.404 5	189.910 7	161.775 4
0.4	242.326 3	220.621 7	197.717 2	173.510 2	147.886 6
0.5	226.876 8	205.793 4	183.627 6	160.294 0	135.697 8
0.6	207.659 0	187.420 3	166.233 6	144.030 8	120.736 8
0.7	185.522 7	166.100 6	145.893 8	124.853 9	102.928 1
0.8	144.503 8	128.566 4	112.080 4	95.017 0	77.345 5
0.9	112.683 1	98.642 6	84.237 3	69.452 7	54.273 7

	When data is generated from Poisson distribution				
0.1	208.198 7	193.633 0	178.182 8	161.765 0	144.286 0
0.2	200.344 9	186.282 4	171.381 0	155.563 5	138.742 6
0.3	189.485 1	176.179 2	162.097 7	147.170 8	131.320 0
0.4	181.498 6	168.589 7	154.945 0	140.499 7	125.181 2
0.5	164.227 9	152.522 1	140.175 9	127.135 1	113.339 6
0.6	152.881 1	141.837 5	130.206 9	117.941 2	104.987 0
0.7	133.280 9	123.706 1	113.648 1	103.069 4	91.928 3
0.8	119.479 0	110.693 3	101.483 1	91.816 9	81.660 1
0.9	91.544 9	84.914 0	77.985 1	70.737 8	63.149 4

**Table 9:** Values of PRE_5_ when *ρ* ∈ (0.1, 0.2, 0.3, 0.4, 0.5, 0.6, 0.7, 0.8, 0.9) and response rates are 75%, 80%, 85%, 90%,  and 95% for data generated from normal, Gamma, and Poisson distributions.

*ρ*	PRE_5_ when response rate is				
	75%	80%	85%	90%	95%
	When data is generated from normal distribution				
0.1	1361.451 1	1484.757 6	1615.473 2	1754.286 6	1901.974 3
0.2	523.041 9	562.926 2	605.004 8	649.463 8	696.511 2
0.3	504.883 7	541.794 7	580.570 3	621.355 7	664.311 1
0.4	998.053 7	1078.963 1	1163.678 3	1252.474 2	1345.653 0
0.5	769.279 0	828.016 2	889.329 7	953.392 7	1020.394 5
0.6	341.143 5	360.152 7	379.861 5	400.309 2	421.538 1
0.7	458.557 7	485.768 8	513.782 2	542.633 9	572.362 1
0.8	354.110 3	373.251 8	393.005 4	413.401 2	434.470 8
0.9	434.663 4	459.058 3	484.088 3	509.778 4	536.155 3

	When data is generated from Gamma distribution				
0.1	226.951 0	239.763 0	253.386 3	267.900 4	283.395 8
0.2	227.539 8	240.069 8	253.361 5	267.486 8	282.526 4
0.3	230.863 6	243.383 3	256.633 4	270.679 9	285.596 7
0.4	224.737 5	236.253 2	248.405 5	261.248 9	274.843 9
0.5	226.091 9	237.201 0	248.880 3	261.175 1	274.135 1
0.6	226.830 4	237.395 3	248.455 0	260.045 2	272.204 9
0.7	227.996 6	237.780 7	247.960 1	258.559 3	269.604 7
0.8	213.751 3	221.499 4	229.514 2	237.809 7	246.400 8
0.9	207.722 6	213.871 4	220.180 0	226.654 7	233.302 1

	When data is generated from Poisson distribution				
0.1	177.014 5	184.577 7	192.600 1	201.124 8	210.200 7
0.2	176.981 0	184.423 1	192.309 1	200.679 9	209.581 7
0.3	176.082 6	183.287 9	190.913 3	198.996 4	207.579 9
0.4	176.690 9	183.813 5	191.342 2	199.312 6	207.764 7
0.5	174.141 1	180.768 9	187.759 3	195.142 9	202.953 8
0.6	173.116 4	179.477 2	186.176 0	193.240 6	200.701 8
0.7	167.633 3	173.193 0	179.033 2	185.175 9	191.645 0
0.8	165.091 7	170.188 2	175.530 9	181.138 2	187.030 1
0.9	153.828 1	157.554 6	161.448 6	165.521 6	169.786 3

**Table 10:** Values of PRE_6_ when *ρ* ∈ (0.1, 0.2, 0.3, 0.4, 0.5, 0.6, 0.7, 0.8, 0.9) and response rates are 75%, 80%, 85%, 90%,  and 95% for data generated from normal, Gamma, and Poisson distributions.

*ρ*	PRE_6_ when response rate is				
	75%	80%	85%	90%	95%
	When data is generated from normal distribution				
0.1	2062.182 7	2061.693 8	2061.175 5	2060.625 1	2060.039 5
0.2	757.255 6	755.313 4	753.264 3	751.099 3	748.808 2
0.3	728.352 1	724.973 9	721.425 0	717.692 2	713.760 8
0.4	1494.992 5	1485.649 2	1475.866 3	1465.612 3	1454.852 1
0.5	1138.649 9	1129.854 3	1120.672 8	1111.079 6	1101.046 4
0.6	471.604 1	466.406 9	461.018 4	455.428 0	449.623 9
0.7	653.137 9	643.694 4	633.972 5	623.959 7	613.642 6
0.8	491.045 6	484.524 4	477.794 5	470.845 9	463.667 7
0.9	615.439 5	605.537 5	595.377 6	584.949 8	574.243 2

	When data is generated from Gamma distribution				
0.1	297.869 3	298.238 8	298.631 7	299.050 2	299.497 1
0.2	298.423 0	298.449 3	298.477 2	298.506 9	298.538 5
0.3	303.239 0	302.924 8	302.592 3	302.239 8	301.865 5
0.4	293.266 4	292.551 0	291.796 2	290.998 4	290.153 9
0.5	294.795 7	293.539 4	292.218 7	290.828 4	289.362 9
0.6	295.282 7	293.410 3	291.450 2	289.396 0	287.241 0
0.7	296.138 8	293.373 9	290.497 3	287.502 1	284.380 7
0.8	273.078 6	269.764 2	266.335 7	262.787 2	259.112 2
0.9	262.412 9	258.184 8	253.846 9	249.394 7	244.823 8

	When data is generated from Poisson distribution				
0.1	219.817 0	219.828 6	219.841 0	219.854 1	219.868 0
0.2	219.610 4	219.514 2	219.412 4	219.304 2	219.189 2
0.3	218.016 2	217.784 9	217.540 0	217.280 5	217.004 9
0.4	218.776 0	218.414 6	218.032 6	217.628 2	217.199 3
0.5	214.476 7	213.896 6	213.284 7	212.638 5	211.954 8
0.6	212.655 0	211.926 9	211.160 0	210.351 3	209.497 3
0.7	203.733 4	202.783 9	201.786 5	200.737 4	199.632 6
0.8	199.461 7	198.332 7	197.149 2	195.907 1	194.601 9
0.9	181.441 7	180.131 0	178.761 4	177.328 8	175.828 7

**Table 11:** Values of PRE_7_ when *ρ* ∈ (0.1, 0.2, 0.3, 0.4, 0.5, 0.6, 0.7, 0.8, 0.9) and response rates are 75%, 80%, 85%, 90%,  and 95% for data generated from normal, Gamma, and Poisson distributions.

*ρ*	PRE_7_ when response rate is				
	75%	80%	85%	90%	95%
	When data is generated from normal distribution				
0.1	2062.182 7	2061.693 8	2061.175 5	2060.625 1	2060.039 5
0.2	757.255 6	755.313 4	753.264 3	751.099 3	748.808 2
0.3	728.352 1	724.973 9	721.425 0	717.692 2	713.760 8
0.4	1494.992 5	1485.649 2	1475.866 3	1465.612 3	1454.852 1
0.5	1138.649 9	1129.854 3	1120.672 8	1111.079 6	1101.046 4
0.6	471.604 1	466.406 9	461.018 4	455.428 0	449.623 9
0.7	653.137 9	643.694 4	633.972 5	623.959 7	613.642 6
0.8	491.045 6	484.524 4	477.794 5	470.845 9	463.667 7
0.9	615.439 5	605.537 5	595.377 6	584.949 8	574.243 2

	When data is generated from Gamma distribution				
0.1	297.869 3	298.238 8	298.631 7	299.050 2	299.497 1
0.2	298.423 0	298.449 3	298.477 2	298.506 9	298.538 5
0.3	303.239 0	302.924 8	302.592 3	302.239 8	301.865 5
0.4	293.266 4	292.551 0	291.796 2	290.998 4	290.153 9
0.5	294.795 7	293.539 4	292.218 7	290.828 4	289.362 9
0.6	295.282 7	293.410 3	291.450 2	289.396 0	287.241 0
0.7	296.138 8	293.373 9	290.497 3	287.502 1	284.380 7
0.8	273.078 6	269.764 2	266.335 7	262.787 2	259.112 2
0.9	262.412 9	258.184 8	253.846 9	249.394 7	244.823 8

	When data is generated from Poisson distribution				
0.1	219.817 0	219.828 6	219.841 0	219.854 1	219.868 0
0.2	219.610 4	219.514 2	219.412 4	219.304 2	219.189 2
0.3	218.016 2	217.784 9	217.540 0	217.280 5	217.004 9
0.4	218.776 0	218.414 6	218.032 6	217.628 2	217.199 3
0.5	214.476 7	213.896 6	213.284 7	212.638 5	211.954 8
0.6	212.655 0	211.926 9	211.160 0	210.351 3	209.497 3
0.7	203.733 4	202.783 9	201.786 5	200.737 4	199.632 6
0.8	199.461 7	198.332 7	197.149 2	195.907 1	194.601 9
0.9	181.441 7	180.131 0	178.761 4	177.328 8	175.828 7

**Table 12:** Values of bias when *ρ* ∈ (0.1, 0.2, 0.3, 0.4, 0.5, 0.6, 0.7, 0.8, 0.9) and response rates are 75%, 80%, 85%, 90%,  and 95% for data generated from normal, Gamma, and Poisson distributions.

*ρ*	Bias of the proposed estimator when response rate is				
	75%	80%	85%	90%	95%
	When data is generated from normal distribution				
0.1	−0.000 095 000 0	−0.000 092 000 0	−0.000 090 000 0	−0.000 088 000 0	−0.000 086 000 0
0.2	−0.000 093 000 0	−0.000 088 000 0	−0.000 083 000 0	−0.000 078 000 0	−0.000 075 000 0
0.3	−0.000 121 000 0	−0.000 114 000 0	−0.000 108 000 0	−0.000 102 000 0	−0.000 097 000 0
0.4	−0.000 114 000 0	−0.000 104 000 0	−0.000 096 000 0	−0.000 089 000 0	−0.000 082 000 0
0.5	−0.000 126 000 0	−0.000 113 000 0	−0.000 101 000 0	−0.000 091 000 0	−0.000 082 000 0
0.6	−0.000 135 000 0	−0.000 120 000 0	−0.000 106 000 0	−0.000 094 000 0	−0.000 083 000 0
0.7	−0.000 143 000 0	−0.000 126 000 0	−0.000 111 000 0	−0.000 098 000 0	−0.000 086 000 0
0.8	−0.000 154 000 0	−0.000 133 000 0	−0.000 115 000 0	−0.000 099 000 0	−0.000 084 000 0
0.9	−0.000 159 000 0	−0.000 136 000 0	−0.000 115 000 0	−0.000 097 000 0	−0.000 081 000 0

	When data is generated from Gamma distribution				
0.1	−0.000 030 580 0	−0.000 030 510 0	−0.000 030 460 0	−0.000 030 400 0	−0.000 030 360 0
0.2	−0.000 026 300 0	−0.000 026 150 0	−0.000 026 020 0	−0.000 025 900 0	−0.000 025 800 0
0.3	−0.000 030 400 0	−0.000 030 190 0	−0.000 030 010 0	−0.000 029 840 0	−0.000 029 690 0
0.4	−0.000 027 760 0	−0.000 027 420 0	−0.000 027 110 0	−0.000 026 840 0	−0.000 026 600 0
0.5	−0.000 027 550 0	−0.000 027 110 0	−0.000 026 730 0	−0.000 026 390 0	−0.000 026 080 0
0.6	−0.000 032 820 0	−0.000 032 170 0	−0.000 031 590 0	−0.000 031 080 0	−0.000 030 620 0
0.7	−0.000 029 350 0	−0.000 028 530 0	−0.000 027 810 0	−0.000 027 170 0	−0.000 026 590 0
0.8	−0.000 031 190 0	−0.000 030 150 0	−0.000 029 230 0	−0.000 028 420 0	−0.000 027 690 0
0.9	−0.000 034 810 0	−0.000 033 330 0	−0.000 032 030 0	−0.000 030 880 0	−0.000 029 850 0

	When data is generated from Poisson distribution				
0.1	−0.000 000 403 0	−0.000 000 395 0	−0.000 000 387 0	−0.000 000 381 0	−0.000 000 375 0
0.2	−0.000 000 447 0	−0.000 000 430 0	−0.000 000 415 0	−0.000 000 402 0	−0.000 000 390 0
0.3	−0.000 000 455 0	−0.000 000 431 0	−0.000 000 411 0	−0.000 000 392 0	−0.000 000 376 0
0.4	−0.000 000 510 0	−0.000 000 473 0	−0.000 000 440 0	−0.000 000 411 0	−0.000 000 385 0
0.5	−0.000 000 552 0	−0.000 000 503 0	−0.000 000 460 0	−0.000 000 421 0	−0.000 000 387 0
0.6	−0.000 000 632 0	−0.000 000 568 0	−0.000 000 511 0	−0.000 000 461 0	−0.000 000 416 0
0.7	−0.000 000 703 0	−0.000 000 620 0	−0.000 000 547 0	−0.000 000 483 0	−0.000 000 425 0
0.8	−0.000 000 777 0	−0.000 000 675 0	−0.000 000 585 0	−0.000 000 505 0	−0.000 000 434 0
0.9	−0.000 000 871 0	−0.000 000 746 0	−0.000 000 635 0	−0.000 000 536 0	−0.000 000 448 0

## Data Availability

The data used in the study are generated theoretically by the equations given in this paper.
